# GntR Family of Bacterial Transcription Factors and Their DNA Binding Motifs: Structure, Positioning and Co-Evolution

**DOI:** 10.1371/journal.pone.0132618

**Published:** 2015-07-07

**Authors:** Inna A. Suvorova, Yuri D. Korostelev, Mikhail S. Gelfand

**Affiliations:** 1 Research and Training Center on Bioinformatics, Institute for Information Transmission Problems RAS (The Kharkevich Institute), Moscow, Russia; 2 Faculty of Bioengineering and Bioinformatics, Moscow State University, Moscow, Russia; National Center for Biotechnology Information, UNITED STATES

## Abstract

The GntR family of transcription factors (TFs) is a large group of proteins present in diverse bacteria and regulating various biological processes. Here we use the comparative genomics approach to reconstruct regulons and identify binding motifs of regulators from three subfamilies of the GntR family, FadR, HutC, and YtrA. Using these data, we attempt to predict DNA-protein contacts by analyzing correlations between binding motifs in DNA and amino acid sequences of TFs. We identify pairs of positions with high correlation between amino acids and nucleotides for FadR, HutC, and YtrA subfamilies and show that the most predicted DNA-protein interactions are quite similar in all subfamilies and conform well to the experimentally identified contacts formed by FadR from *E*. *coli* and AraR from *B*. *subtilis*. The most frequent predicted contacts in the analyzed subfamilies are Arg-G, Asn-A, Asp-C. We also analyze the divergon structure and preferred site positions relative to regulated genes in the FadR and HutC subfamilies. A single site in a divergon usually regulates both operons and is approximately in the middle of the intergenic area. Double sites are either involved in the co-operative regulation of both operons and then are in the center of the intergenic area, or each site in the pair independently regulates its own operon and tends to be near it. We also identify additional candidate TF-binding boxes near palindromic binding sites of TFs from the FadR, HutC, and YtrA subfamilies, which may play role in the binding of additional TF-subunits.

## Introduction

Interactions between DNA and proteins lie at the heart of many biological processes including DNA recombination, replication, repair and transcription [1]. One of the main mechanisms of regulation of gene expression is specific binding of transcription factors (TFs) to DNA. While up to 10% of genes in genomes of free-living bacteria encode transcription factors [2, 3], their structure and DNA-binding specificity are usually unknown [1]. Understanding the recognition mechanism of protein-DNA interaction is one of the most important problems of molecular and computational biology. Evolution of regulatory interactions in various organisms can be studied by comparative analysis of functional systems.

Empirical rules of the protein-DNA recognition reflect chemical and physical properties of the residues, such as partial charge interactions between amino acid side chains and bases, or amino acid side chain flexibility [4]. The contribution of the amino acid main chain to the specific interaction with DNA is minor compared to the amino-acid side-chain atoms [5], and important and favorable contacts are usually hydrogen bonds (because of their high specificity and directional character) and acid—base interactions [4, 6, 7]. However, they do not always dominate in determining the interaction, and other types of contacts are also important [8]. For example, though hydrophobic interactions are considered less important for DNA-binding, since there are relatively few non-polar atoms present in the DNA double helix grooves [4], and regions of protein-DNA contacts are rich in polar residues that are important for binding, as they are involved in the formation of electrostatic and hydrogen bonds [9], hydrophobic interactions can play a certain role in protein-DNA interaction. While hydrogen bonds are specific in recognizing purines, hydrophobic contacts are mainly involved in recognition of the pyrimidines, for example, protein side chains rely on hydrophobic interactions to differentiate thymine from cytosine [10].

However, these trends are not universal and do not explain all amino acid—base interactions that may depend on the structural context and, in particular, on the structural family of DNA-binding proteins [10, 11].

Conservation of base pairs in a motif is significantly correlated with the number of contacts they have with the bound TF [5, 8]. Base pairs that form more contacts tend to be more conserved in evolution, because some of these amino acid-base pair interactions stabilize the DNA-protein complex and changes in these positions are more deleterious [8]. Mutual information analysis can be used to predict amino acid—base contacts for particular transcription factor families, giving opportunity to yield structural insights from sequence information alone, which can be further experimentally verified [12–16].

Contacts between the protein and the DNA sugar-phosphate backbone are thought to play a minor role in determining the specificity [10], but they may impact it by positioning of TF recognition elements in an orientation allowing for proper interaction [10, 17].

### GntR family

The GntR family of transcription factors, first described in 1991 and named after the gluconate-operon repressor in *Bacillus subtilis*, is a large group of proteins distributed among diverse bacteria and regulating various biological processes [18, 19, 20]. GntR-family regulatory proteins are comprised of a DNA-binding domain and a signaling domain, linked together [19, 20, 21]. All proteins from the GntR family share highly similar N-terminal HTH (helix—turn—helix) DNA-binding domains, but differ in the C-terminal effector-binding and oligomerization (E-O) domains [18, 20]. The HTH domain is widespread and detected in many TFs, being the most-studied and best-characterized DNA-binding motif in the prokaryotic world [18, 20, 21]. The N-terminal DNA-binding domain of GntR-family proteins comprises a central β-sheet cluster and three α-helices [20]. The HTH motif consists of the α-helix, the connecting loop, and the second α-helix, often referred to as the “recognition helix”, as it directly interacts with the DNA [18, 20, 22]. Generally, HTH proteins bind as dimers to 2-fold symmetric DNA operator sequences so that each monomer recognizes a half-site [20, 22].

The C-terminal E-O domain does not bind to the DNA, but it can impose steric constraints on the DNA-binding domain, hence influencing the HTH motif, and thus plays an important role in regulation [21]. For example, E-O domain can restrict DNA-binding domain’s mobility and thus reduce its ability to adapt to varying distances between the parts of a palindromic motif, which reflects on the binding motif structure [20]. Oligomerization and conformational changes due to binding of an inducer molecule allow for the correct HTH motif arrangement, modulating its orientation and presentation, and the subsequent DNA binding [20, 21], as shown for many diverse proteins [23, 24]. Thus, despite high conservation of the DNA-binding domain, the operator consensus sequences observed among GntR-family TFs may be different, likely due to the E-O domain variability and domain synergy [20].

According to the type of the C-terminal domain, the GntR family is divided into four main (FadR, HutC, MocR, and YtrA) and two minor subfamilies (AraR and PlmA) [18, 20, 21, 25–29].

The FadR subfamily is the largest one, it comprises about 40% of known GntR-family TFs, with α-helical C-terminal domain, which is 150–170 amino acids in length, formed by either seven or six α-helices [18, 20]. TFs of the FadR subfamily bind effectors, small organic ligands, such as carboxylic acids, and then undergo conformational changes that affect DNA-binding [18]. Most FadR-subfamily proteins are involved in the regulation of oxidized substrates related to amino acids or emerging from the central metabolism, or at the crossroads of various metabolic pathways, such as glycolate (GlcC), galactonate (DgoR), pyruvate (PdhR), lactate (LldR), or gluconate (GntR) [18, 20].

The C-terminal domain of the second subfamily, HutC, is about 170 amino acids in length and contains both α-helical and β-sheet structures [20]. This subfamily comprises about 30% of GntR-family regulators [20]. The C-terminal E-O domain of HutC-subfamily transcription factors has the same fold as chorismate lyase (UbiC in *Escherichia coli*), which suggests that it may bind small effector molecules, such as histidine (HutC), fatty acids (FarR), sugars (TreR), and alkylphosphonates (PhnF), in a mode similar to chorismate lyase [19]. Some HutC-subfamily TFs are involved in the regulation of N-acetylglucosamine utilization (DasR, NagR, NagQ) and in conjugative plasmid transfer in various *Streptomyces* species (e.g., KorSA, KorA, and TraR) [20, 22, 30].

The third subfamily, MocR, is different, as proteins from this group have a large E-O domain, whose average length is about 350 amino acids [20]. This domain is homologous to class I aminotransferase proteins (TyrB of *E*. *coli*) [20, 31, 32]. The latter catalyze transamination of amino acids to α-keto acids and use pyridoxal 5’-phosphate (PLP) as a cofactor [20, 31, 32]. A similar requirement for PLP was shown for some MocR-subfamily proteins (TauR, GabR) [25, 31, 32]; moreover, PdxR in *Streptomyces* spp. is directly involved in the regulation of the PLP synthesis [20, 33]. Aminotransferases are known to form head-to-tail dimers and such dimerization likely occurs in MocR-subfamily proteins as well [20, 32].

Proteins from the fourth subfamily, YtrA, which is the smallest one among the main subfamilies (about 6%), have a reduced C-terminal domain with only two α-helices, of the average length about 50 amino acids [20]. This may seriously restrict effector-binding and dimerization abilities of the C-terminal domain, though the latter is obviously possible, since long palindromic binding motifs have been identified upstream of candidate regulated operons [20]. Most genes of the YtrA-subfamily TFs form operons with ATP-binding cassette (ABC) transporters [20].

The PlmA subfamily is composed exclusively of TFs from cyanobacterial species [26]. It is close to the YtrA and MocR subfamilies, and its likely ancestor arose from one of them [26]. PlmA (encoded by *all1076*) controls plasmid maintenance in *Anabaena* (*Nostoc*) sp. strain PCC 7120, but it is unclear whether it is a common function of PlmA-subfamily TFs, since there are no identified plasmids in several cyanobacterial species, all of which contain *plmA* orthologs [26].

AraR-subfamily TFs exhibit chimeric organization with two domains of different phylogenetic origin: its N-terminal DNA-binding region contains a winged HTH motif similar to that of the GntR family, while the C-terminal domain is homologous to the C-terminal domain of the GalR/LacI family [27, 28, 29]. AraR controls expression of genes encoding transporters and enzymes involved in uptake and utilization of L-arabinose and arabinose-containing polysaccharides, xylose and galactose in Firmicutes [27, 28, 29].

### Structure of binding motifs

Different DNA-binding domain types recognize distinct motifs [34], whereas DNA-binding proteins from the same family tend to recognize sites of similar length, symmetry, and specificity [5]. Within each family, structure and fold of the DNA-binding domain and its mode of interaction with the binding motif are usually conserved, which results in a characteristic pattern of DNA-amino acid contacts [5]. However, even proteins with very high (up to 60–70%) amino acid sequence identity may bind to distinct DNA motifs [34].

As mentioned above, the HTH motifs are conserved in all the GntR family, although there are differences between consensus sequences for each subfamily [20]. The level of similarity between the HTH domains of the MocR and YtrA subfamilies is the highest. One of these two subfamilies has likely emerged from the other via replacement of the C-terminal domain [20].

Many experimentally identified and predicted binding motifs of GntR-family TFs match the palindromic N_y_GTN_x_ACN_y_ consensus sequence [20]. The motifs differ in the number (x, y) and the nature (N) of the nucleotides that surround the consensus GT and AC pairs [20]. This neighborhood often consists of A and T residues, and their number differs between subfamilies [20]. For example, the consensus for the FadR-subfamily TFs is N_y_GTM-N_0–1_-KACN_y_, and for the HutC subfamily, N_y_GTMTAKACN_y_ [20,21]. The center of the palindrome is usually highly conserved, while the periphery varies [20].

Some TFs from the FadR and HutC subfamilies recognize unique motifs with different or no symmetry [20], for example, FarR (direct repeats TGTATTAWTT) [35], NagQ (direct repeats TGGTATT) [30], BioR (TTATMKATAA) [36, 37], NanR (direct repeats TGGTATAW) [38].

The distance between the half-sites is important for the correct presentation of a DNA site to a TF, and it varies weakly among the FadR and HutC subfamilies, but differs between these groups and the YtrA subfamily [20]. In the YtrA subfamily, the conserved GT and AC residues are located far from the center of the palindrome [20]. This feature of motifs may be due to short C-terminal domains of the YtrA-subfamily TFs, which may cause a specific mode of dimerization and DNA-binding, and hence yield an unusual motif structure [20].

Comparative studies of the MocR subfamily did not reveal any conserved palindromic sequence satisfying the GntR consensus or common to the whole subfamily [20]. For example, predicted binding motifs for some of the MocR-subfamily regulatory proteins include direct repeats of ATACCA for GabR [31], CTGGACYTAA for TauR [25] and AAAGTGGW(−/T)CTA for PdxR [39]. There are no obvious similarities in these structures and thus they may not be compared. Such organization of binding motifs could be due to the head-to-tail dimerization of MocR-type TFs, which yields direct repeats with sufficiently long spacers that allow for DNA looping [20].

Several crystal structures of GntR-family proteins have been solved so far, for example, in the FadR subfamily, these are FadR from *Escherichia coli* (PDB code 1H9T, 1HW1, 1HW2), LldR from *Corynebacterium glutamicum* (2DI3), TM0439 from *Thermotoga maritime* (3SXK, 3SXY); in the HutC subfamily, YvoA (NagR) from *Bacillus subtilis* (2WV0), HutC from *Pseudomonas syringae pv*. *tomato* str. DC3000 (2PKH), AgaR from *Enterococcus faecalis* V583 (3DDV); and in the AraR subfamily, AraR DNA-binding domain from *Bacillus subtilis* (4EGY, 4EGZ, 4H0E). However, only two of these TFs (FadR and AraR) are solved in a complex with DNA. The structural data (summarized in Table 1) shows that FadR from *E*. *coli* and AraR from *B*. *subtilis* form a number of non-specific interactions with the DNA sugar-phosphate backbone, but only few base pairs are specifically recognized within the complex [40, 41, 42]. Main base-specific interaction present in FadR-DNA complex and in all analyzed structures of AraR DNA-binding domain with DNA is a hydrogen bond formed by Arg, which is part of the recognition helix, with the base G [28, 40, 41, 42]. Thus, such recognition may be a conserved feature of the GntR family.

**Table 1:** DNA-amino acid contacts in FadR from *E*.*coli* and AraR from *B*. *subtilis*.

Position in the HTH domain	Amino acid of FadR *E*.*coli*	Contact in FadR-DNA or other related function	Amino acid of AraR *B*. *subtilis*	Contact in AraR-DNA or other related function
0	Ser-7	Non-specific with sugar-phosphate backbone	Pro-25	-
1	Pro-8	Non-specific with sugar-phosphate backbone	Lys-26	Non-specific with sugar-phosphate backbone
2	Ala-9	Non-specific with sugar-phosphate backbone	Tyr-27	Non-specific with sugar-phosphate backbone
27	Glu-34	Non-specific with sugar-phosphate backbone; electrostatic bonds with Arg-35, Arg-45, Arg-49	Glu-52	Hydrogen bonds with Arg-63, Arg-67
28	Arg-35	Arg-G, specific	Asn-53	-
37	Thr-44	Non-specific with sugar-phosphate backbone; Thr-C and Thr-G, specific	Ser-62	-
38	Arg-45	Arg-G, specific	Arg-63	Arg-G, specific; Arg-A, water-mediated specific; Arg-A, acetate-mediated
39	Thr-46	Non-specific with sugar-phosphate backbone; Thr-C and Thr-G, specific	His-64	His-G and His-T, water-mediated specific
40	Thr-47	Non-specific with sugar-phosphate backbone	Thr-65	Non-specific with sugar-phosphate backbone
42	Arg-49	Non-specific with sugar-phosphate backbone	Arg-67	Non-specific with sugar-phosphate backbone
43	Glu-50	Electrostatic bonds with Arg-35, Arg-45, Arg-49	Lys-68	-
56	Ile-63	Non-specific with sugar-phosphate backbone	Ser-81	-
58	His-65	His-A and His-G, specific	Gln-83	Gln-A and Gln-T, specific
59	Gly-66	Non-specific with sugar-phosphate backbone; helps avoiding steric clash	Gly-84	Gly-T, specific; Gly-T and Gly-A, water-mediated specific; Gly-T and Gly-A, acetate-mediated; helps avoiding steric clash
60	Lys-67	Non-specific with sugar-phosphate backbone	Gly-85	-
62	Thr-69	Non-specific with sugar-phosphate backbone	Gly-86	-

### Goals

We use the comparative genomics approach to reconstruct regulons and predict binding motifs of the regulators from three subfamilies of the GntR family—FadR, HutC, and YtrA. We report correlations between the DNA binding motifs and amino acid sequences of TFs and predict the most favorable DNA-protein contacts.

Further, we analyze the divergon structure in the FadR and HutC subfamilies and characterize preferred site positions relative to regulated genes.

We also identify additional candidate TF-binding boxes near strong binding sites in a number of the orthologous groups of transcription factors from the FadR, HutC, and YtrA subfamilies.

## Materials and Methods

All genomic sequences were obtained from GenBank [43]. Known GntR-family TFs were collected from the literature. New members of the family were found using exhaustive BLAST search [44]. Homologs of TFs were found by PSI-BLAST [44] searches (E-value cutoff, 10^−20^), and orthologs were identified by construction of phylogenetic trees for identified homologs supplemented by analysis of gene neighborhoods on chromosomes (*e*.*g*., co-localization with genes of a certain metabolic pathway in most genomes). Normally, an ortholog group contained one TF per genome. However, in some cases several TFs in one genome, resulting from recent duplications or close-range horizontal transfers, were assigned to the same ortholog group.

Amino acid and nucleotide sequence alignment was performed using the MUSCLE package (with default parameters) [45]. Phylogenetic trees were constructed with the PHYLIP package, using the protdist program for the calculation of distances and the maximum-likelihood method for the tree construction (with default parameters) [46].

Candidate binding sites were identified (or confirmed if they were previously predicted) by phylogenetic footprinting [2]. We manually analyzed alignments of upstream regions of orthologous genes and identified groups of consecutive conserved positions, relying on the assumption that binding sites are more conserved than surrounding intergenic regions. Nucleotide position weight matrices (PWMs, profiles) for each TF were then constructed by the SignalX program as previously described [47], using training sets of upstream regions of genes presumably belonging to the respective regulon (genes encoding TFs, as they are often auto-regulated, and genes co-localized with them). The profiles were then used to search for additional regulon members.

Computational search for candidate TF-binding sites in upstream gene regions (for all genes in genomes, 400 nucleotides (nt) upstream and 50 nt downstream relative to the annotated gene start) was performed using the GenomeExplorer program package [48] and the RegPredict web server [49]. Score thresholds for the identification of sites were selected so that candidate sites upstream of functionally relevant genes were accepted, while the fraction of genes preceded by candidate sites did not exceed 5% in each studied genome. Under these conditions, for some long, conserved motifs, the number of candidate sites per genome did not exceed 50. Weaker sites (with scores 10% less than the threshold) were also taken into account if their positions were similar to positions of stronger sites upstream of orthologous genes and there were no stronger competing sites in the same intergenic region. New candidate members were assigned to a regulon if they were preceded by candidate binding sites in several genomes, the exact number of genomes depending on the number of sequenced genomes in a taxonomy unit. The reconstructed regulons were extended to include all genes in putative operons, the latter defined as the strings of genes transcribed in the same direction, with intergenic distances not exceeding 200 nt, when such organization persisted in several genomes. Motif logos were constructed using WebLogo [50].

Data on composition of the characterized GntR-family regulons, and respective binding sites are available in RegPrecise database (http://regprecise.lbl.gov/RegPrecise/).

Only TFs that had palindromic predicted binding motifs satisfying the GntR-family consensus were selected to analyze the correlation between amino acid sequences of TFs’ DNA-binding HTH domains and nucleotides in the binding sites. The structural data of FadR from *Escherichia coli* and AraR from *Bacillus subtilis* in complexes with DNA was taken as a reference model. At that, positions of known interacting amino acids [40, 41, 42] were re-numbered starting from the beginning of the HTH domain, counting from zero (Table 1).

Correlations were calculated for each subfamily using the Prot-DNA-Korr program package. The program calculates the correlation between each pair of columns, one from the amino acid alignment, and the other from the site alignment (dataset used in this work is given in S1 File). As a measure of correlation, the mutual information is used. The statistical significance value of the mutual information is calculated as the Z-score. Correlated pairs of positions are presented as a heatmap, where the pairs are colored according to the statistical significance, and as the contingency tables (given in S2 File) which contain expected and observed counts of amino acid-nucleotide pairs. For more detailed information concerning Prot-DNA-Korr program, see the link: http://bioinf.fbb.msu.ru/Prot-DNA-Korr/main.html.

Statistical analysis was performed using the STATISTICA program package [51].

The comparative genomic analysis relied on the following basic assumptions. It is known that the majority (~60–70%) of bacterial TFs are auto-regulated [52, 53]. Negative auto-regulation, in which a transcription factor represses its own gene expression, is the most common network motif (e.g., approximately 40% of the known TFs in *E*. *coli*) [54, 55]. Besides auto-regulation, in many cases genes encoding TFs and the genes they regulate are co-localized in the genome, since they tend to evolve concurrently, and this fact can be used when linking novel TFs with their DNA motifs and candidate regulon members [47,53].

## Results and Discussion

Here we report the results of the analysis of transcription factors from three subfamilies of the GntR family—FadR, HutC, and YtrA. Candidate binding sites were predicted for 1252 GntR-family TFs from 307 genomes of bacterial species (S1 Table). The TFs were classified into 64 orthologous groups. The representation of the GntR-family TFs in individual genomes and among taxonomic groups varied (Table 2). For example, YtrA-subfamily TFs are common among *Firmicutes*, while FadR-subfamily TFs are more typical for *Proteobacteria*.

**Table 2:** General statistics of the analyzed GNTR-family TFs.

Number of \ Subfamily		FadR	HutC	YtrA
Orthologous groups		36	16	12
TFs analysed		634	389	229
Regulated operons, total		1740	975	283
Sites total (including divergent and multiple)		2396	1341	294
**Taxonomy distribution of analyzed TFs**				
Proteobacteria	Alpha	76	39	3
	Beta	151	64	0
	Gamma	308	112	25
	Delta	10	1	0
Firmicutes	Bacilli	18	97	89
	Clostridia	1	14	53
Actinobacteria		64	60	43
Thermotogae		0	0	14
Chloroflexi		6	0	1
Cyanobacteria		0	1	0
Bacteroidetes		0	1	0
Archaea		0	0	1

### Protein-DNA correlations

#### FadR subfamily

**Fig 1:**
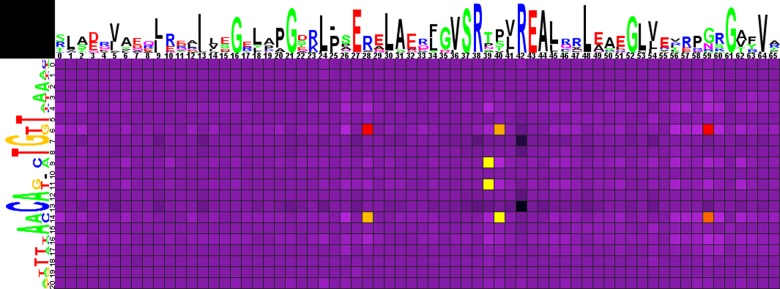
Heat map of correlations between amino acids and nucleotides for FadR-subfamily TFs and their binding sites. Sequence logos of HTH DNA-binding domains and corresponding binding sites are shown on the top and to the left of the heat map, respectively. The total height of the symbols in each position equals the positional information content, whereas the height of individual symbols is proportional to the positional amino acid or nucleotide frequency. The correlation scores are color ramped from yellow to red for amino acid-nucleotide pairs with statistical significance greater than an automatically defined threshold (with red assigned for the most correlated pair). The violet-black palette is used for other pairs.

The common consensus of all analyzed binding sites of FadR-subfamily TFs is an A/T-rich palindromic sequence with conserved TKGT/ACMA boxes (Fig 1), likely the most important for the DNA-protein interaction. As was mentioned earlier, the typical distance between GT and AC in most FadR-subfamily TF-binding sites is 3 nt (DgoR, ExuR, FadR, GlcC, LldR, PdhR *etc*), although in several orthologous groups it is 2 nt (*e*.*g*., GntR, HpxS, HypR, MdcY, PrpR, UxuR). The latter sites were included into the dataset (Spreadsheet FadR in S1 File) for further analysis after insertion of a single-nucleotide gap into the center of the motif.

Some FadR-subfamily TFs were excluded from the correlation analysis, since their binding motifs did not conform to the common consensus sequence and hence could not be aligned. The examples are NanR that binds direct repeats TGGTATAW [38], or BioR with the binding site consensus TTATMKATAA [36,37].

The correlation analysis (Fig 1, Spreadsheet FadR in S2 File) shows that, for FadR-subfamily TFs, significant amino acid positions correlated with the site positions and likely responsible for the binding specificity correspond well to those identified for the FadR (*E*.*coli*) and AraR (*B*.*subtilis*) protein-DNA structures.

Due to the symmetrical structure of the analyzed binding motifs and, consequently, of the obtained heat maps, correlations are usually shown for either G/C or A/T pair, while further disambiguation between G and C, or A and T is not always possible, since it requires additional consideration, such as comparing correlation data to the known contacts in the FadR-DNA and AraR-DNA complexes, taking into account donor-acceptor properties etc. It is known that, in general, hydrogen-bond donor residues (like Arg, His, Lys, Ser, Thr) prefer G, hydrogen bond acceptor residues (such as acidic Asp, Glu) prefer C, while Asn and Gln, that possess both donor and acceptor moieties, prefer A [6, 7].

Amino acids in position 28 of the HTH domain are correlated with nucleotides in site positions 6/14 (in TKGT/ACMA groups), known to form a contact in FadR-DNA complex (Table 1) [40, 41]. Arg, the most frequent amino acid in this position, strongly prefers the G/C pair, while the A/T pair is significantly avoided. Asp, which is much rarer in position 28 than Arg, also significantly prefers the G/C pair. According to the electrochemical characteristics of these amino acids, we can conclude that the possible contacts in this position are Arg-G and Asp-C.

Amino acid residues in positions 40 and 59, which are known to be important for the FadR and AraR interactions with DNA (Table 1), are also correlated with nucleotide in positions 6/14. The most frequent amino acids in position 40 are Pro and Ser. Ser significantly prefers the G/C pair (possibly interacting with G), while Pro significantly avoids it.

Gly, that is most frequent in position 59, strongly prefers the G/C pair, while the A/T pair is significantly avoided. However, the Gly-G/C association might not be linked to a direct contact. In FadR-DNA complex, glycine occupying the same position does not form specific contacts, but due to the absence of the side chain allows for the interaction of the adjacent amino acid with DNA [40, 41]. Asn is also frequent in position 59 and exhibits a preference of the A/T pair, but it is not statistically significant.

Moreover, amino acids in position 39 of the HTH domain, also involved in binding to DNA by FadR and AraR (Table 1), are correlated with central nucleotides in positions 9/11. Asn here significantly prefers the A/T pair, possibly interacting with A according to the interaction trends described above. Thr is also frequent in this position and it shows a trend towards the preference of the A/T pair, but it is not statistically significant.

#### HutC subfamily

**Fig 2:**
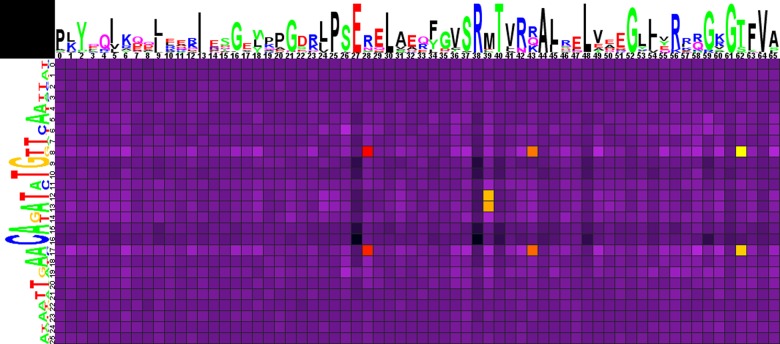
Heat map of correlations between amino acids and nucleotides for HutC-subfamily TFs and their binding sites. Notation as in Fig 1.

The consensus sequence of all analyzed binding motifs of HutC-subfamily TFs is very similar to the one of the FadR subfamily (Fig 2). The distance between GT and AC in most HutC-subfamily TF binding sites is 4 nt. Among the exceptions there are FarR (direct repeats TGTATTAWTT) [35], NagQ (direct repeats TGGTATT) [30], SdhR (palindrome with additional symmetry TCTTATGTCTTATATAAGACATAAGA) [56]. These TFs were excluded from the correlation analysis, as their binding sites could not be aligned and compared with the main group of sites (Spreadsheet HutC in S1 File).

The correlation analysis (Fig 2, Spreadsheet HutC in S2 File) shows that positions significant for binding specificity of HutC-subfamily TFs resemble the ones identified for FadR from *E*.*coli* and the FadR subfamily in general. In particular, amino acids in position 28 of the HTH domain correlate with nucleotides 8/17. As in the FadR subfamily, Arg, the most frequent amino acid in position 28, strongly prefers the G/C pair (according to the electrochemical characteristics, possible contact in this position is Arg-G), while the A/T pair is significantly avoided. Asn is also frequent in this position of the HTH domain, weakly preferring the A/T pair (no statistical significance).

Amino acid residues in positions 43 and 62 also show correlations with nucleotides in positions 8/17. The most frequent amino acids are Arg, Gln, Lys in position 43, and Thr and Ser in position 62, but neither of them shows significant preference of any base pair, while less frequent in position 62 Trp significantly prefers the G/C pair (possibly interacting with C).

Moreover, amino acids in position 39 of the HTH domain are correlated with central nucleotides 12/13 (as it has been shown for the FadR subfamily). The most frequent amino acid in this position is Met, with non-significant preference of the A/T pair, while less frequent Asp here significantly prefers the G/C pair, where it likely interacts with C, since it is a hydrogen bond acceptor amino acid.

#### YtrA subfamily

This subfamily, its binding motifs and regulons have many features different from those of other studied GntR subfamilies. The divergent organization of regulated operons, frequently observed for FadR- and HutC-subfamilies TFs (see below), is very rare in YtrA-subfamily regulons. Consistent with previous observations [20], most YtrA-subfamily regulons consist of a single operon comprised of genes encoding ATP-binding cassette (ABC) transporters. Moreover, most genes regulated by YtrA-subfamily TFs are preceded by single binding sites, and very few double or triple binding sites (quite common for the FadR and HutC subfamilies) have been identified (Spreadsheet YtrA in S1 File).

**Fig 3:**
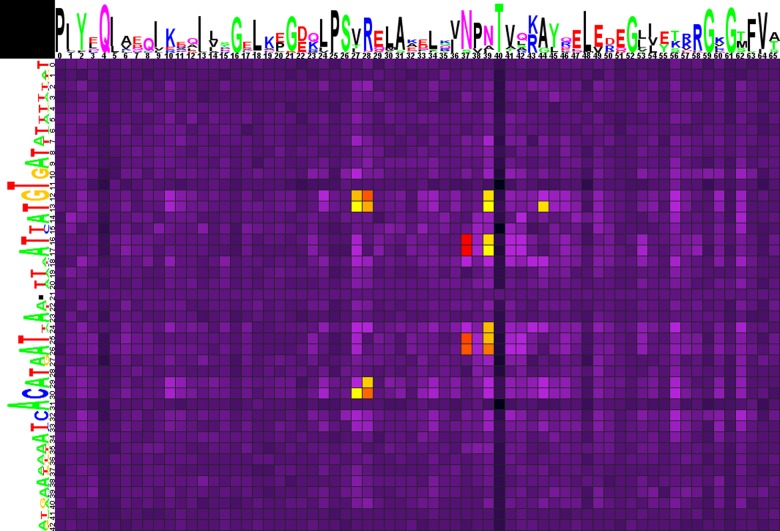
Heat map of correlations between amino acids and nucleotides for YtrA-subfamily TFs and their binding sites. Notation as in Fig 1.

Binding motifs of TFs from the YtrA subfamily are significantly longer than motifs of other GntR-family TFs (Fig 3). Still, due to the conserved HTH domain structure in the GntR family, YtrA-type DNA-binding domains can be aligned accurately with domains from the other subfamilies, and our analysis has shown that amino acid positions that determine the binding specificity in the YtrA subfamily are mostly similar to those of FadR and HutC.

As was already mentioned, consensuses of the GntR-family binding motifs are generally palindromic, but each particular site can deviate from the consensus, being not strictly symmetric. In the case of FadR and HutC subfamilies these deviations are averaged by the large number of studied sites (Table 2), and thus the corresponding correlation data heat maps are symmetric. The YtrA subfamily is the smallest one, with the number of analyzed sites being an order of magnitude less than in other subfamilies (Table 2), which leads to some asymmetries in the corresponding correlation data heat map (Fig 3). Moreover, asymmetry can be caused by the lack of the divergently regulated operons in YtrA subfamily, unlike the FadR and HutC subfamilies.

The correlations (Fig 3, Spreadsheet YtrA in S2 File) show that nucleotides in positions 12-13/29-30 may specifically interact with amino acids in positions 27 and 28. As in the case of FadR and HutC subfamilies, Arg and G/C is the most frequent amino acid/nucleotide pair in position 28, though they do not show significant correlation here, while Asn and Tyr, rarer in this position, are significantly associated with the A/T pair (both likely interacting with A, according to the interaction trends described above). Val is the most frequent amino acid in position 27, but it shows no statistically significant base-pair preferences, while Thr, that is also frequent here, significantly prefers the A/T pair (possibly interacting with A, being a polar uncharged amino acid) in nucleotide positions 12,13 and 30.

Correlations are also observed for nucleotides 16-17/25-26 and amino acids 37 and 39, and that conforms well to the structural data for FadR, where these positions are important for the interaction with DNA (Table 1). Asn is the most frequent amino acid residue in positions 37 and 39, weakly preferring the A/T pair in 16-17/25-26 positions (no statistical significance). Ser, less common in position 37, and Ile in position 39, both strongly prefer A/T in positions 16-17/25-26 of the motif; while His in position 39 is significantly correlated with the G/C pair in positions 25 and 26. In the latter case the contact is likely His-G, conforming to the interaction trends of the hydrogen-bond donor residues, as well as to the His-G contact in the same position in the AraR-DNA complex (Table 1).

Moreover, significant correlations are also identified for Ala in position 39 with A/T in nucleotide positions 12, 13 and with G/C in position 24; and Gly in position 44 is correlated with A/T in position 13.

Overall, despite significant differences in the binding motifs, DNA-protein interactions in the YtrA subfamily seem to be at least partly similar to that of the FadR and HutC subfamilies.

#### Overview of protein-DNA correlations

Our data shows that predicted protein-DNA interactions for all three analyzed subfamilies of the GntR family correspond well to known nucleotide-amino acid contacts of FadR and AraR [40, 41, 42].

It has been shown in the literature that Arg, Asn, Lys, Gln, Thr, Ser, Asp and Gly account for more than 70% of contacts, with Arg alone accounting for 23% [7]. This trend was demonstrated in our study as well: majority of the predicted interactions involved exactly these amino acids.

Arg-G, Asn-A, Asp-C, Gln-A, Glu-C, Lys-G, and to a lesser extent His-G and Ser-G, appear to be the most relevant, strongest and highly specific contacts [4, 7]. Preferences are also known for Ala-C, Cys-G, Gly-G, Leu-A, Thr-G, and Trp-C [7].

Though there is some controversial data (for example, both Ser-A/T and Ser-G/C correlations), the majority of favorable contacts (Arg-G, Asn-A, Asp-C, Gly-G, His-G, Trp-C), predicted by the correlation analysis of the GntR-family TFs and their binding sites in all analyzed subfamilies, conforms to the general interaction trends described in the literature [6, 7].

### Divergons

Many genes regulated by FadR—and HutC-subfamily TFs are organized in two divergently transcribed operons (divergons), and it is not immediately clear what is the relationship between the intergenic sites and each of the operons. The YtrA subfamily has not been represented in this analysis, as TFs from this subfamily almost never have sites between divergently transcribed operons.

The divergons were divided into two groups: divergons that consist of structural genes only (the control group), and divergons comprising a TF gene. Divergons with single or double intergenic sites were studied separately.

For divergons with a single binding site, we analyzed the length of the intergenic region and the distance between the center of the binding site and the starts of both genes that form the divergon.

For divergons with double binding sites we calculated the length of the intergenic region, the distance between the center of the proximal binding site and the start of a gene, and the distance between the two binding-sites’ centers.

In the case of divergons with single sites, our aim was to determine whether these sites regulate both divergent operons, or one operon only (e.g., divergon could comprise a regulated operon containing structural genes and a divergent, not auto-regulated TF gene).

It is known that TFs, for example AraR, can cooperatively bind to several adjacent sites, allowing for a more flexible and tight control of the expression [42, 57]. In the case of divergons with double sites, we aimed to distinguish between the following alternatives: the site pair could be involved in regulation of the both divergent operons (or one particular operon), hence essentially being a single complex site, or each site in the pair could separately regulate its own operon.

#### Divergons with a single site

**Fig 4:**
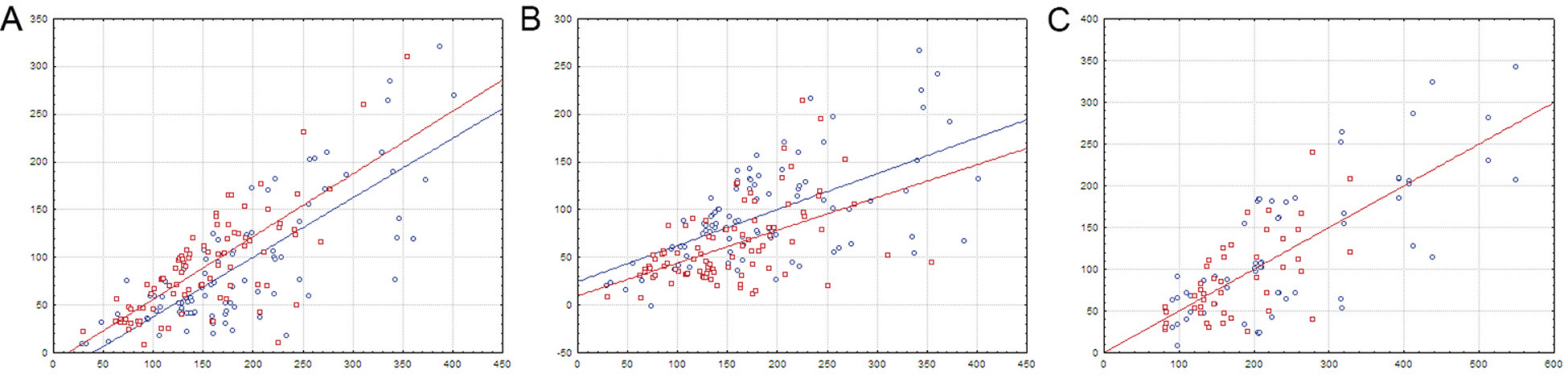
Distances between regulated genes and TF-binding sites in divergons with single sites. A—operons with a TF gene; B—operons with structural genes only; C—the control group (includes divergons without TF genes). The vertical axis is the distance between the site center and the start codon. The horizontal axis is the intergenic distance. Each dot corresponds to one site. The regression lines are shown. Blue color denotes the FadR subfamily; red color, the HutC subfamily.

For both FadR- (n = 96) and HutC-subfamily (n = 94) divergons comprising a TF gene in one of the operons we observed an approximately linear increase of the distance between the site and the start of each gene in the divergon, as the intergenic distance increased (Fig 4A and 4B). The same tendency was also observed for the control divergons (FadR, n = 33; HutC, n = 23) (Fig 4C; due to the complete match only one regression line is visible). Thus, single sites usually tend to be localized approximately in the middle of the intergenic spacer, although in the divergons with TF genes they usually are slightly closer to the structural operon (Table 3, Fig 4).

**Table 3:** Interdependence of the intergenic distance and the distance to a single site.

Linear regression coefficient (R^2^)			
	Both subfamilies	FadR	HutC
Operons with a TF gene	0,60 (0,55)	0,62 (0,60)	0,66 (0,56)
Operons with structural genes only	0,40 (0,35)	0,38 (0,35)	0,34 (0,26)
Control divergons	0,50 (0,58)	0,50 (0,58)	0,50 (0,42)
Pearson correlation coefficient (p-value)			
	Both subfamilies	FadR	HutC
Operons with a TF gene	0,74 (p<1·10^−7^)	0,77 (p<1·10^−7^)	0,75 (p<1·10^−7^)
Operons with structural genes only	0,59 (p<1·10^−7^)	0,59 (p<1·10^−7^)	0,51 (p = 2·10^−7^)
Control divergons	0,76 (p<1·10^−7^)	0,76 (p<1·10^−7^)	0,65 (p = 1·10^−6^)

#### Divergons with double sites

As mentioned earlier, there are three possible variants of regulation in the case of divergons with double sites. If each site in a pair regulates its own operon, the distance between the sites should be positively correlated with the size of the intergenic region, as each site would tend to be closer to its regulated operon. Vice versa, if the sites are co-operatively involved in the regulation of the both operons (or one particular operon from a pair), the distance between the sites would likely be approximately constant and hence would not correlate with the size of the intergenic region. In this case, similarly to the single-site one, if the common site pair is involved in the regulation of both divergent operons, these sites would tend to be situated in the central part of intergenic region, otherwise, the site pair would be positioned near the regulated operon.

**Fig 5:**
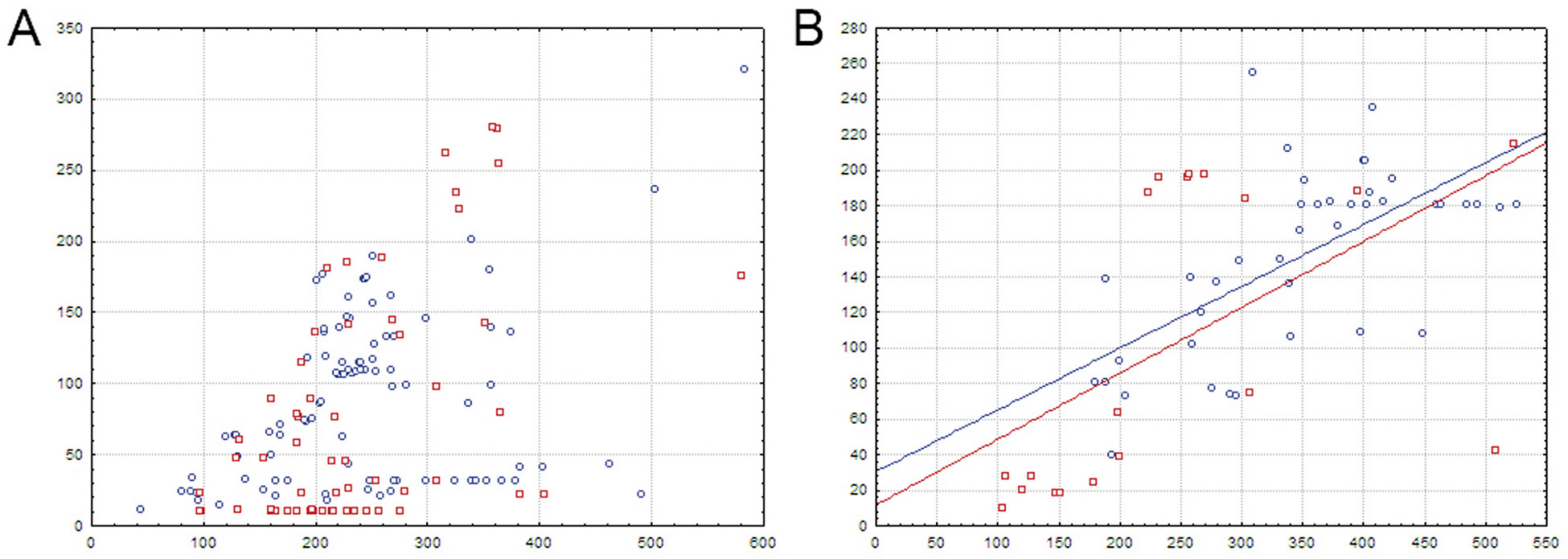
Distances between double sites in divergons. A—divergons with a TF gene; B—the control group. The vertical axis is the inter-site distance. The horizontal axis is the intergenic distance. Notation as in Fig 4.

**Fig 6:**
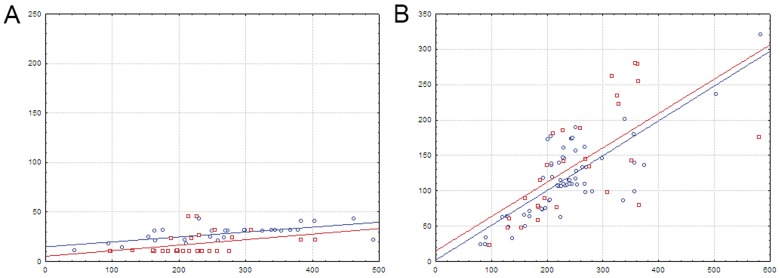
Two groups of divergons with double sites. A—the first group (constant inter-site distance); B—the second group (increasing inter-site distance). For details, see the text. The vertical axis is the inter-site distance. The horizontal axis is the intergenic distance. Notation as in Fig 4.

**Fig 7:**
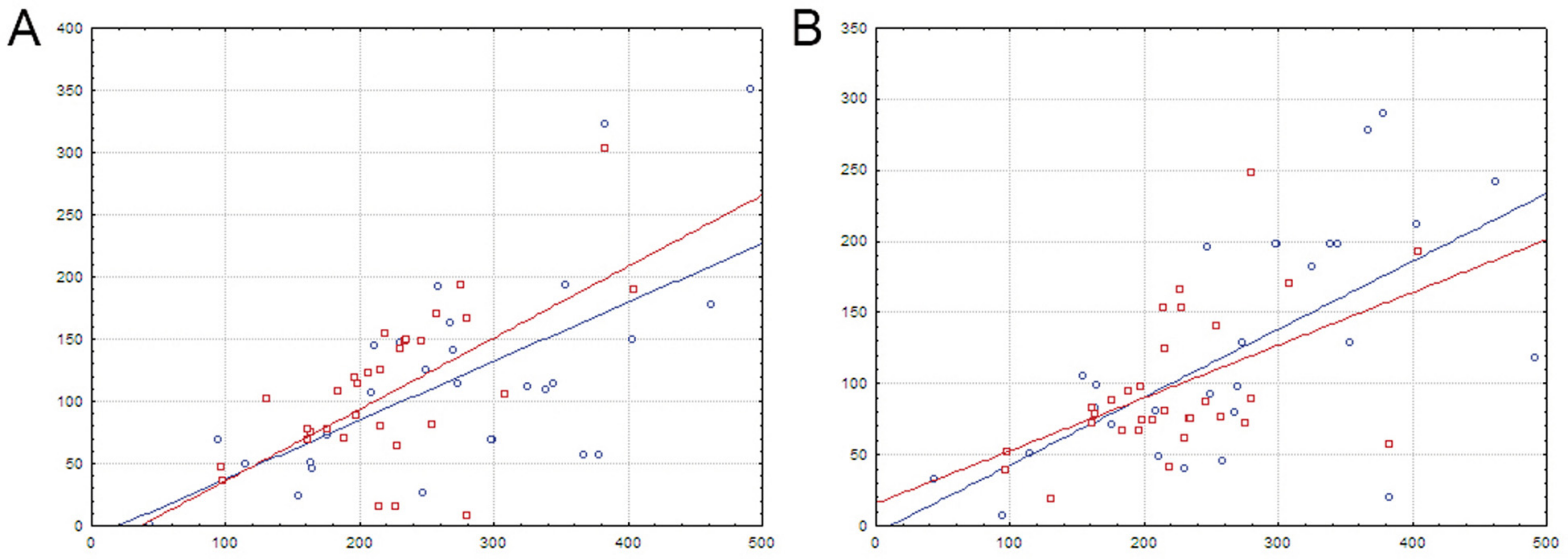
Distances between regulated genes and proximal TF-binding sites in divergons with common double sites. A—operons with a TF gene; B—operons with structural genes only. The vertical axis is the distance between the site center and the start codon. The horizontal axis is the intergenic distance. Notation as in Fig 4.

In both FadR (n = 100) and HutC (n = 60) subfamilies, we observe two fractions of divergons with a TF gene (Table 4, Fig 5A). The first group includes divergons (FadR, n = 29; HutC, n = 32) where the distance between double sites is relatively constant (Fig 6A). In this group, the distance to the proximal binding site tends to be higher for longer intergenic regions in both structural operons and operons with a TF gene (Table 5, Fig 7A and 7B). Thus, double sites in this group of divergons usually tend to be localized near the center of the intergenic area, and we may conclude that they form a pair involved in the co-operative control of both operons.

**Table 4:** Two fractions of divergons with double sites, interdependence of the intergenic distance and the distance between double sites.

Linear regression coefficient (R^2^)			
	Both subfamilies	FadR	HutC
Divergons with common sites (constant inter-site distance)	0,06 (0,26)	0,05 (0,41)	0,06 (0,11)
Divergons with separate sites (increasing inter-site distance)	0,50 (0,53)	0,49 (0,61)	0,49 (0,43)
Pearson correlation coefficient (p-value)			
	Both subfamilies	FadR	HutC
Divergons with common sites (constant inter-site distance)	0,51 (p = 3·10^−5^)	0,64 (p = 2·10^−4^)	0,32 (p = 0,07)
Divergons with separate sites (increasing inter-site distance)	0,73 (p<1·10^−7^)	0,78 (p<1·10^−7^)	0,65 (p = 2·10^−4^)

**Table 5:** Interdependence of the intergenic distance and the distance to the proximal TF-binding site in divergons with common double sites.

Linear regression coefficient (R^2^)			
	Both subfamilies	FadR	HutC
Operons with a TF gene	0,48 (0,38)	0,47 (0,40)	0,57 (0,37)
Operons with structural genes only	0,45 (0,38)	0,48 (0,42)	0,37 (0,24)
Pearson correlation coefficient (p-value)			
	Both subfamilies	FadR	HutC
Operons with a TF gene	0,62 (p = 1·10^−7^)	0,63 (p = 2·10^−4^)	0,61 (p = 2·10^−4^)
Operons with structural genes only	0,62 (p = 1·10^−7^)	0,64 (p = 2·10^−4^)	0,49 (p = 4·10^−3^)

**Fig 8:**
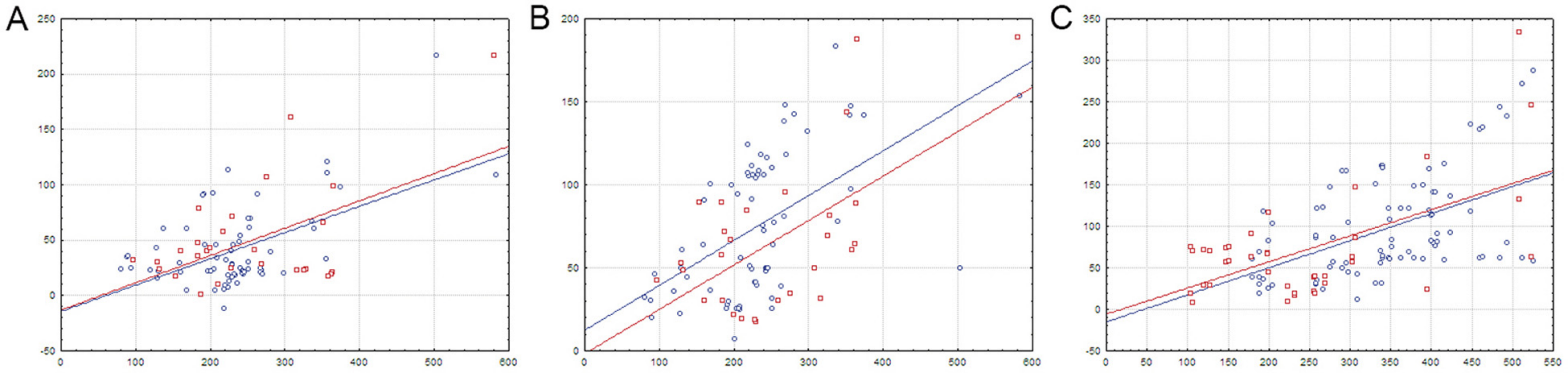
Distances between regulated genes and proximal TF-binding sites in divergons with separate double sites. A—operons with a TF gene; B—operons with structural genes only; C—the control group. The vertical axis is the distance between the site center and the start codon. The horizontal axis is the intergenic distance. Notation as in Fig 4.

The second group (FadR, n = 71; HutC, n = 28) consists of divergons where the distance between the sites in a pair linearly increases with the size of the intergenic region (Fig 6B). Thus, these sites are presumably independent, and each of them controls its own operon. There is also a trend towards increasing of the distance to the proximal site for both structural operons and operons with a TF gene, as the intergenic region grows, but this trend is not as prominent as in case of divergons with relatively constant inter-site distance (the first group) (Table 6, Fig 8A and 8B). The same tendencies were also observed in the control group in both FadR (n = 46) and HutC (n = 19) subfamilies (Table 6, Figs 5B and 8C). Thus, in the control group there is only one type of divergons, where the sites do not act co-operatively, and each likely regulates the adjacent operon.

**Table 6:** Interdependence of the intergenic distance and the distance to the proximal TF-binding site in divergons with separate double sites.

Linear regression coefficient (R^2^)			
	Both subfamilies	FadR	HutC
Operons with a TF gene	0,24 (0,29)	0,24 (0,29)	0,25 (0,29)
Operons with structural genes only	0,26 (0,29)	0,27 (0,27)	0,27 (0,37)
Control divergons	0,31 (0,31)	0,33 (0,25)	0,31 (0,34)
Pearson correlation coefficient (p-value)			
	Both subfamilies	FadR	HutC
Operons with a TF gene	0,54 (p = 7·10^−9^)	0,54 (p = 2·10^−6^)	0,54 (p = 3·10^−3^)
Operons with structural genes only	0,54 (p = 1·10^−8^)	0,52 (p = 3·10^−6^)	0,61 (p = 6·10^−4^)
Control divergons	0,56 (p<1·10^−7^)	0,50 (p = 3·10^−7^)	0,58 (p = 1·10^−4^)

### Additional half-sites near binding sites of the GntR-family TFs

A typical GntR-family binding motif is a palindrome, but we have found that considerable number of identified palindromic binding sites is accompanied by a weaker adjacent half-site (box) at a distance of 7–12 nt. For a more quantitative analysis, regions flanking candidate binding sites of all studied TFs were considered. In 23 analyzed orthologous groups (13 groups, 170 TFs and 450 binding sites in the FadR subfamily; 4 groups, 186 TFs and 514 sites in the HutC subfamily; and 6 groups, 120 TFs and 167 binding sites in the YtrA subfamily; data not shown) weaker boxes were found at the 7–12 nt distance from the binding site center (on one or both sides of the site). These additional boxes and their positions relative to the center of the binding motif were initially identified by visual analysis of logo diagrams of all aligned binding sites and their neighborhood for TFs forming orthologous groups.

**Fig 9:**
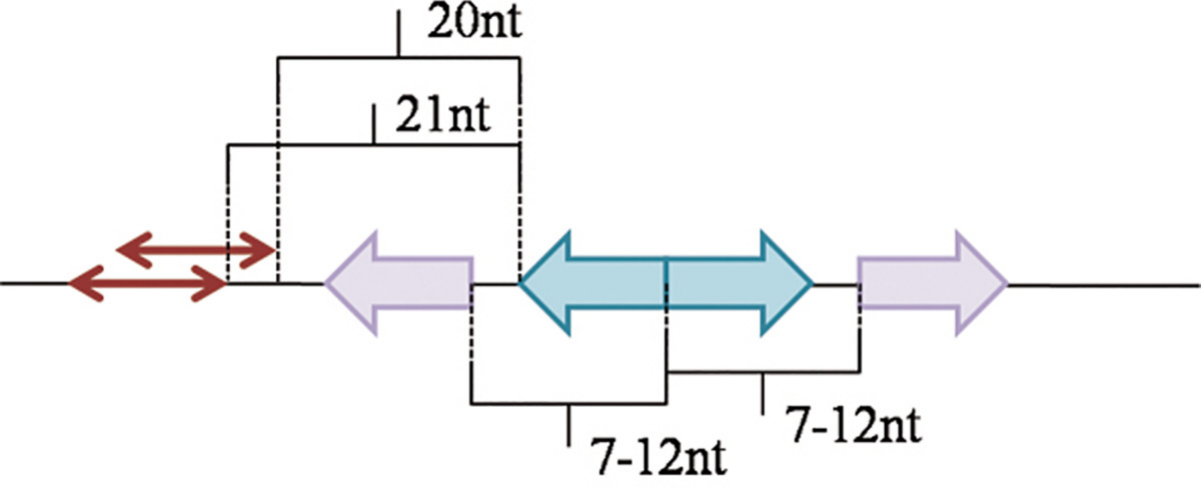
Positioning of additional boxes and control pseudoboxes. True binding half-sites are shown in blue; additional boxes, in violet; pseudoboxes, in red arrows. For details, see the text.

To estimate the significance of this observation, additional boxes were compared to the boxes forming true sites and to the random sequences (pseudoboxes, as control) of the same length. The latter were taken from positions −20 and −21 nt from the binding site (Fig 9). Two pseudoboxes per each binding site were selected to allow for correct estimation of the statistical significance (see below).

The score for each half of a true palindromic site was calculated using the corresponding part of the PWM for this TF (W_true left_ and W_true right_, respectively). The same partial PWMs were used to calculate the scores of additional boxes (W_near left_ and W_near right_, respectively) and pseudoboxes. The score for each pseudobox was calculated twice using the left and right partial PWMs (W_random left1,2_ and W_random right1,2_). Additional boxes with the larger score from each pair (W_near left_ or W_near right_) were selected for further analysis. At that, each additional box was compared with the higher scoring one of two pseudoboxes in the same orientation (left or right).

The length and structure of binding motifs and thus PWMs among various orthologous groups of TFs differ, and hence the calculated scores could not be directly compared. To account for the diversity of motifs, all scores were normalized to the respective W_true_ values for half-sites in the similar orientation as each given additional box:

$$S_{near}=\frac{W_{true}-W_{near}}{W_{true}}$$ (1)

$$S_{random}=\frac{W_{true}-W_{random}}{W_{true}}$$ (2)

where S_near_ and S_random_ denote normalized weights for additional boxes and pseudoboxes, respectively; W_true_, W_near_, W_random_ are respectively the weights of the true half-sites, additional boxes and pseudoboxes, calculated using PWM for the respective TF.

**Fig 10:**
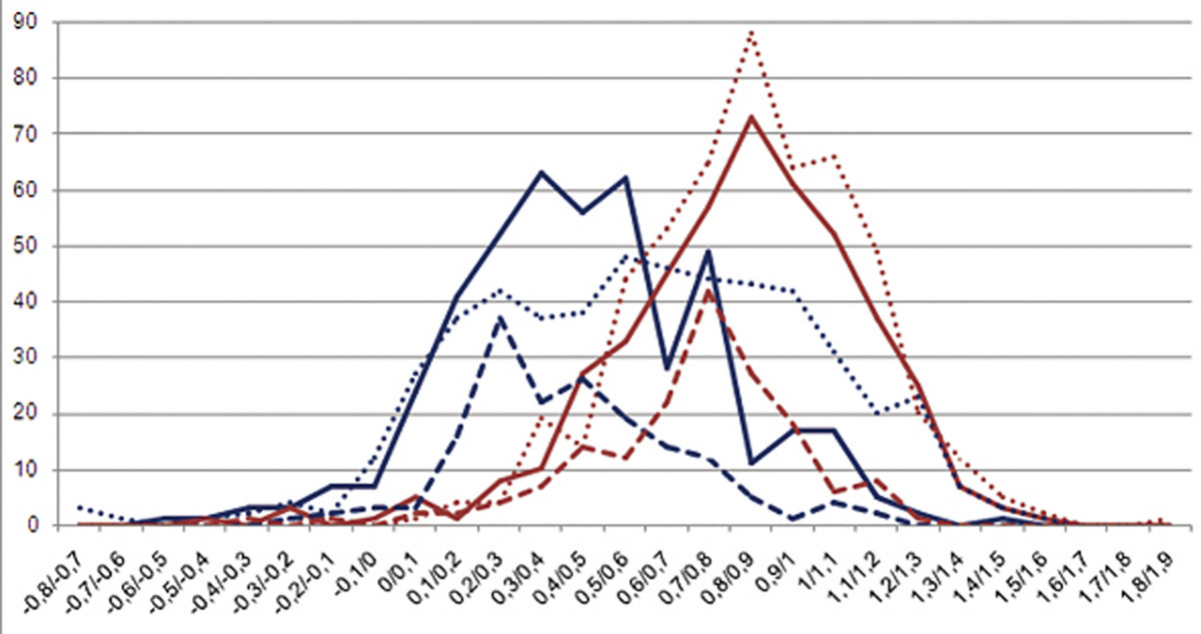
Distribution of S_near_ and S_random_ in the FadR, HutC and YtrA subfamilies. The vertical axis—the number of S values falling in the given interval. The horizontal axis—intervals of S values. Blue color denotes S_near_ values; red color—S_random_ values. FadR subfamily data is shown in continuous lines; HutC subfamily, in dotted lines; YtrA subfamily, in dashed lines.

The distributions of S_near_ and S_random_ (Fig 10) were significantly different for all three subfamilies, FadR, HutC, and YtrA (the Wilcoxon rank-sum test, p<0.001). Moreover, the average W_near_ value approximately equals half of the average W_true_ value, while average W_random_ value is close to zero, confirming that the used control is correct. These boxes may play a role in the regulation, though their exact function should be a subject of further experimental study.

## Conclusions

In this work we identify regulated genes and binding sites for 1252 GNTR-family TFs from the 64 orthologous groups and three subfamilies, FadR, HutC, and YtrA. Using these data, we predict most favorable DNA-protein contacts by analysis of the correlations between amino acids of the TFs and nucleotides of the corresponding binding motifs. Correlation analysis shows that, despite significant differences in the structure of TFs from different subfamilies, main predicted contacts (Arg-G, Asn-A, Asp-C *etc*) are quite similar and conform well to the DNA-protein contacts known for FadR from *E*. *coli* and AraR from *B*. *subtilis*, as well as to the interaction trends described in literature.

Apart from identifying usual palindromic binding sites of GntR-family TFs, we also demonstrate that these motifs may sometimes be extended by additional boxes. They may possibly be involved in alternative TF dimerization, or participate in recruiting additional subunits of TFs, their oligomerization and co-operative regulation, thus allowing for the more flexible and precise transcription control.

Analysis of the divergon structure in the FadR and HutC subfamilies revealed some tendencies in the site localization. A single site in a divergon is usually positioned approximately in the middle of the intergenic area and thus may regulate both operons. It is also interesting to note that for divergons with a TF gene, distance between the single binding site and the structural operon increases slower than the distance to the operon comprising a TF gene, as the intergenic distance grows. This might reflect the fact that TF auto-regulation is slightly weaker than regulation of the corresponding structural genes. Double sites are presumably either involved in the cooperative regulation of both operons and are localized in the center of the intergenic area, or each site in the pair independently regulates its own operon and tends to be near it. Thus we classify dual binding sites in divergons into co-operative and operon-specific ones. Unfortunately, we do not find any functional differences between these two types of divergons, since there is no evident distinction in their gene content.

## Supporting Information

S1 Table Genome abbreviations. (DOC)

S1 File GntR-family TFs, corresponding binding sites and regulated operons. Datasets for the correlation analysis.
(XLS)

S2 File Contingency tables of amino acid-nucleotide pairs. Pairs which occur significantly more often than expected are colored red (strongly preferred) and yellow, those occurring less often than expected are colored blue (strongly avoided).
(XLS)
