# Supplementary material for: GntR Family of Bacterial Transcription Factors and Their DNA Binding Motifs: Structure, Positioning and Co-Evolution
Source: PLoS One. 2015 Jul 7;10(7):e0132618. doi: 10.1371/journal.pone.0132618 (PMC4494728; doi:10.1371/journal.pone.0132618)
Supplement: S1 Table — (DOC) [file pone.0132618.s001.doc]

S1 Table. Genome abbreviations

| Genome | Abbreviation |
| --- | --- |
| Acidiphilium cryptum JF-5 | ACR |
| Acidothermus cellulolyticus 11B | ACE |
| Acidovorax avenae subsp. citrulli AAC00-1 | AAV |
| Acinetobacter baumannii AYE | ABY |
| Acinetobacter sp. ADP1 | ACI |
| Actinobacillus pleuropneumoniae AP76 | APA |
| Actinobacillus succinogenes 130Z | ASU |
| Actinosynnema mirum DSM 43827 | AMR |
| Aeromonas hydrophila subsp. hydrophila ATCC 7966 | AHA |
| Aeromonas salmonicida subsp. salmonicida A449 | ASA |
| Agrobacterium tumefaciens C58 | ATU |
| Alcanivorax borkumensis SK2 | ABO |
| Aliivibrio salmonicida LFI1238 | VSA |
| Alkalilimnicola ehrlichei MLHE-1 | AEH |
| Alkaliphilus metalliredigens QYMF | AMT |
| Alkaliphilus oremlandii OhILAs | AOE |
| Alteromonas macleodii 'Deep ecotype' | AMC |
| Anoxybacillus flavithermus WK1 | AFL |
| Arthrobacter aurescens TC1 | AAU |
| Arthrobacter sp. FB24 | ART |
| Azoarcus sp. BH72 | AZO |
| Azorhizobium caulinodans ORS 571 | AZC |
| Azotobacter vinelandii AvOP | AVI |
| Bacillus amyloliquefaciens FZB42 | BAY |
| Bacillus anthracis Ames | BAN |
| Bacillus cereus ATCC 14579 | BCE |
| Bacillus cereus E33L | BCZ |
| Bacillus clausii KSM-K16 | BCL |
| Bacillus halodurans C-125 | BHA |
| Bacillus licheniformis ATCC 14580 | BLI |
| Bacillus pumilus SAFR-032 | BPU |
| Bacillus subtilis subtilis 168 | BSU |
| Bacillus thuringiensis Al Hakam | BTL |
| Bacteroides thetaiotaomicron VPI-5482 | BTH |
| Beutenbergia cavernae DSM 12333 | BCV |
| Bordetella avium 197N | BAV |
| Bordetella bronchiseptica RB50 | BBR |
| Bordetella parapertussis 12822 | BPA |
| Bordetella petrii DSM 12804 | BPT |
| Bradyrhizobium japonicum USDA110 | BJA |
| Bradyrhizobium sp. BTAi1 | BBT |
| Brucella abortus 9-941 (biovar 1) | BMB |
| Brucella canis ATCC 23365 | BCS |
| Brucella melitensis 16M | BME |
| Brucella ovis ATCC 25840 | BOV |
| Brucella suis 1330 | BMS |
| Burkholderia ambifaria MC40-6 | BAC |
| Burkholderia cenocepacia J2315 | BCJ |
| Burkholderia cepacia AMMD | BAM |
| Burkholderia mallei ATCC 23344 | BMA |
| Burkholderia multivorans ATCC 17616 (JGI) | BMU |
| Burkholderia phymatum STM815 | BPH |
| Burkholderia phytofirmans PsJN | BPY |
| Burkholderia pseudomallei 1710b | BPM |
| Burkholderia sp. 383 | BUR |
| Burkholderia thailandensis E264 | BTE |
| Burkholderia vietnamiensis G4 | BVI |
| Burkholderia xenovorans LB400 | BXE |
| Caldicellulosiruptor saccharolyticus DSM 8903 | CSC |
| Carboxydothermus hydrogenoformans Z-2901 | CHY |
| Catenulispora acidiphila DSM 44928 | CAF |
| Caulobacter crescentus CB15 | CCR |
| Caulobacter sp. K31 | CAK |
| Cellvibrio japonicus Ueda107 | CJA |
| Chloroflexus aggregans DSM 9485 | CAG |
| Chloroflexus aurantiacus J-10-fl | CAU |
| Chloroflexus sp. Y-400-fl | CHS |
| Chromobacterium violaceum ATCC 12472 | CVI |
| Chromohalobacter salexigens DSM 3043 | CSA |
| Citrobacter koseri ATCC BAA-895 | CKO |
| Clavibacter michiganensis michiganensis NCPPB 382 | CMI |
| Clostridium acetobutylicum ATCC 824 | CAC |
| Clostridium beijerinckii NCIMB 8052 | CBE |
| Clostridium botulinum F | CBF |
| Clostridium difficile 630 | CDF |
| Clostridium kluyveri DSM 555 | CKL |
| Clostridium novyi NT | CNO |
| Clostridium perfringens 13 | CPE |
| Clostridium phytofermentans ISDg | CPY |
| Clostridium tetani E88 | CTC |
| Clostridium thermocellum ATCC 27405 | CTH |
| Colwellia psychrerythraea 34H | CPS |
| Conexibacter woesei DSM 14684 | CWO |
| Corynebacterium aurimucosum ATCC 700975 | CAR |
| Corynebacterium diphtheriae gravis NCTC13129 | CDI |
| Corynebacterium efficiens YS-314 | CEF |
| Corynebacterium glutamicum ATCC 13032 | CGB |
| Corynebacterium glutamicum R | CGT |
| Corynebacterium jeikeium K411 | CJK |
| Corynebacterium urealyticum DSM 7109 | CUR |
| Cupriavidus taiwanensis LMG 19424 | CTI |
| Dechloromonas aromatica RCB | DAR |
| Delftia acidovorans SPH-1 | DAC |
| Desulfitobacterium hafniense Y51 | DSY |
| Desulfotomaculum reducens MI-1 | DRM |
| Desulfovibrio desulfuricans G20 | DDE |
| Desulfovibrio vulgaris vulgaris Hildenborough | DVU |
| Dinoroseobacter shibae DFL 12 | DSH |
| Edwardsiella tarda EIB202 | ETD |
| Enterobacter sakazakii ATCC BAA-894 | ESA |
| Enterobacter sp. 638 | ENT |
| Enterococcus faecalis V583 | EFA |
| Erwinia amylovora ATCC 49946 | EAM |
| Erwinia carotovora atroseptica SCRI1043 | ECA |
| Erwinia pyrifoliae Ep1/96 | EPY |
| Erwinia tasmaniensis Et1/99 | ETA |
| Escherichia coli K-12 MG1655 | ECO |
| Escherichia fergusonii ATCC 35469 | EFE |
| Fervidobacterium nodosum Rt17-B1 | FNO |
| Finegoldia magna ATCC 29328 | FMA |
| Frankia alni ACN14a | FAL |
| Frankia sp. EAN1pec | FRE |
| Geobacillus kaustophilus HTA426 | GKA |
| Geobacillus thermodenitrificans NG80-2 | GTN |
| Geobacter metallireducens GS-15 | GME |
| Geobacter sulfurreducens PCA | GSU |
| Geobacter uraniumreducens Rf4 | GUR |
| Gluconacetobacter diazotrophicus PAl 5 | GDI |
| Gluconobacter oxydans 621H | GOX |
| Haemophilus ducreyi 35000HP | HDU |
| Haemophilus influenzae 86-028NP | HIT |
| Haemophilus somnus 129PT | HSO |
| Hahella chejuensis KCTC 2396 | HCH |
| Halothermothrix orenii H 168 | HOR |
| Hyphomonas neptunium ATCC 15444 | HNE |
| Idiomarina loihiensis L2TR | ILO |
| Jannaschia sp. CCS1 | JAN |
| Jonesia denitrificans DSM 20603 | JDE |
| Kineococcus radiotolerans SRS30216 | KRA |
| Klebsiella pneumoniae subsp. pneumoniae MGH 78578 | KPN |
| Kosmotoga olearia TBF 19.5.1 | KOL |
| Kribbella flavida DSM 17836 | KFL |
| Lactobacillus acidophilus NCFM | LAC |
| Lactobacillus brevis ATCC 367 | LBR |
| Lactobacillus casei ATCC 334 | LCA |
| Lactobacillus delbrueckii subsp. bulgaricus ATCC 11842 | LDB |
| Lactobacillus gasseri ATCC 33323 | LGA |
| Lactobacillus johnsonii NCC 533 | LJO |
| Lactobacillus plantarum WCFS1 | LPL |
| Lactobacillus sakei 23K | LSA |
| Lactococcus lactis subsp. cremoris SK11 | LLC |
| Leifsonia xyli subsp. xyli CTCB07 | LXX |
| Leptothrix cholodnii SP-6 | LCH |
| Leuconostoc mesenteroides ATCC 8293 | LME |
| Listeria innocua Clip11262 | LIN |
| Listeria monocytogenes EGD-e | LMO |
| Listeria welshimeri SLCC5334 | LWE |
| Lysinibacillus sphaericus C3-41 | LSP |
| Macrococcus caseolyticus JCSC5402 | MCC |
| Magnetospirillum magneticum AMB-1 | MAG |
| Mannheimia succiniciproducens MBEL55E | MSU |
| Maricaulis maris MCS10 | MMR |
| Marinobacter aquaeolei VT8 | MAQ |
| Marinomonas sp. MWYL1 | MMW |
| Mesorhizobium loti MAFF303099 | MLO |
| Mesorhizobium sp. BNC1 | MES |
| Methanocorpusculum labreanum Z | MLA |
| Methylibium petroleiphilum PM1 | MPT |
| Methylobacterium chloromethanicum CM4 | MCH |
| Methylobacterium extorquens PA1 | MEX |
| Methylobacterium populi BJ001 | MPO |
| Methylobacterium radiotolerans JCM2831 | MRD |
| Methylobacterium sp. 4-46 | MET |
| Methylocella silvestris BL2 | MSL |
| Moorella thermoacetica ATCC 39073 | MTA |
| Mycobacterium avium subsp. paratuberculosis k10 | MPA |
| Mycobacterium bovis BCG Pasteur 1173P2 | MBB |
| Mycobacterium smegmatis MC2 155 | MSM |
| Mycobacterium sp. JLS | MJL |
| Mycobacterium tuberculosis H37Rv | MTU |
| Mycobacterium vanbaalenii PYR-1 | MVA |
| Nakamurella multipartita DSM 44233 | NMU |
| Natranaerobius thermophilus JW/NM-WN-LF | NTH |
| Neisseria meningitidis MC58 | NME |
| Nocardia farcinica IFM 10152 | NFA |
| Nocardioides sp. JS614 | NCA |
| Nostoc sp. PCC 7120 | ANA |
| Novosphingobium aromaticivorans DSM 12444 | NAR |
| Oceanobacillus iheyensis HTE831 | OIH |
| Ochrobactrum anthropi ATCC 49188 | OAN |
| Oenococcus oeni PSU-1 | OOE |
| Oligotropha carboxidovorans OM5 | OCA |
| Paenibacillus sp. JDR-2 | PJR |
| Paracoccus denitrificans PD1222 | PDE |
| Pasteurella multocida PM70 | PMU |
| Pediococcus pentosaceus ATCC 25745 | PPE |
| Pelotomaculum thermopropionicum SI | PTH |
| Petrotoga mobilis SJ95 | PMO |
| Phenylobacterium zucineum HLK1 | PZU |
| Photobacterium profundum SS9 | PPR |
| Photorhabdus luminescens subsp. laumondii TTO1 | PLU |
| Polaromonas naphthalenivorans CJ2 | PNA |
| Polaromonas sp. JS666 | POL |
| Propionibacterium acnes KPA171202 | PAC |
| Proteus mirabilis HI4320 | PMR |
| Pseudoalteromonas atlantica T6c | PAT |
| Pseudoalteromonas haloplanktis TAC125 | PHA |
| Pseudomonas aeruginosa PA01 | PAE |
| Pseudomonas entomophila L48 | PEN |
| Pseudomonas fluorescens Pf-5 | PFL |
| Pseudomonas fluorescens PfO-1 | PFO |
| Pseudomonas mendocina ymp | PMY |
| Pseudomonas putida GB-1 | PPG |
| Pseudomonas putida KT2440 | PPU |
| Pseudomonas putida W619 | PPW |
| Pseudomonas stutzeri A1501 | PSA |
| Pseudomonas syringae pv. tomato DC3000 | PST |
| Psychrobacter arcticum 273-4 | PAR |
| Psychromonas ingrahamii 37 | PIN |
| Ralstonia eutropha H16 | REH |
| Ralstonia eutropha JMP134 | REU |
| Ralstonia metallidurans CH34 | RME |
| Ralstonia pickettii 12J | RPI |
| Ralstonia solanacearum GMI1000 | RSO |
| Rhizobium etli CFN 42 | RET |
| Rhizobium etli CIAT 652 | REC |
| Rhizobium leguminosarum bv. viciae 3841 | RLE |
| Rhodobacter sphaeroides 2.4.1 | RSP |
| Rhodococcus sp. RHA1 | RHA |
| Rhodoferax ferrireducens DSM 15236 | RFR |
| Rhodospirillum centenum SW | RCE |
| Rhodospirillum rubrum ATCC 11170 | RRU |
| Roseiflexus castenholzii DSM13941 | RCA |
| Roseiflexus sp. RS-1 | RRS |
| Roseobacter denitrificans OCh 114 | RDE |
| Rubrobacter xylanophilus DSM 9941 | RXY |
| Saccharophagus degradans 2-40 | SDE |
| Saccharopolyspora erythraea NRRL 2338 | SER |
| Salinispora tropica CNB-440 | STP |
| Salmonella enterica serovar Typhi CT18 | STY |
| Salmonella typhimurium LT2 | STM |
| Serratia proteamaculans 568 | SPE |
| Shewanella amazonensis SB2B | SAZ |
| Shewanella baltica OS155 | SBL |
| Shewanella denitrificans OS217 | SDN |
| Shewanella frigidimarina NCIMB 400 | SFR |
| Shewanella halifaxensis HAW-EB4 | SHL |
| Shewanella loihica PV-4 | SLO |
| Shewanella oneidensis MR-1 | SON |
| Shewanella pealeana ATCC 700345 | SPL |
| Shewanella piezotolerans WP3 | SWP |
| Shewanella putrefaciens CN-32 | SPC |
| Shewanella sediminis HAW-EB3 | SSE |
| Shewanella sp. ANA-3 | SHN |
| Shewanella woodyi ATCC51908 | SWD |
| Shigella boydii Sb227 | SBO |
| Shigella dysenteriae Sd197 | SDY |
| Shigella flexneri 2a str. 301 | SFL |
| Shigella sonnei Ss046 | SSN |
| Sinorhizobium medicae WSM419 | SMD |
| Sinorhizobium meliloti 1021 | SME |
| Sodalis glossinidius morsitans | SGL |
| Sphaerobacter thermophilus DSM 20745 | SPT |
| Sphingomonas wittichii RW1 | SWI |
| Sphingopyxis alaskensis RB2256 | SAL |
| Staphylococcus aureus subsp. aureus Mu50 | SAV |
| Staphylococcus capitis SK14 | SCP |
| Staphylococcus carnosus subsp. carnosus TM300 | SCA |
| Staphylococcus epidermidis ATCC 12228 | SEP |
| Staphylococcus haemolyticus JCSC1435 | SHA |
| Staphylococcus lugdunensis HKU09-01 | SLG |
| Staphylococcus saprophyticus subsp. saprophyticus ATCC 15305 | SSP |
| Stenotrophomonas maltophilia R551-3 | SMT |
| Streptococcus agalactiae 2603V/R | SAG |
| Streptococcus equi subsp. zooepidemicus MGCS10565 | SEZ |
| Streptococcus mutans UA159 | SMU |
| Streptococcus pneumoniae TIGR4 | SPN |
| Streptococcus pyogenes M1 GAS | SPY |
| Streptococcus sanguinis SK36 | SSA |
| Streptococcus suis 05ZYH33 | SSU |
| Streptococcus thermophilus LMD-9 | STE |
| Streptococcus uberis 0140J | SUB |
| Streptomyces avermitilis MA-4680 | SMA |
| Streptomyces coelicolor A3(2) | SCO |
| Streptomyces griseus subsp. griseus NBRC 13350 | SGR |
| Symbiobacterium thermophilum IAM 14863 | STH |
| Syntrophomonas wolfei subsp. wolfei str. Goettingen | SWO |
| Thauera sp. MZ1T | TMZ |
| Thermoanaerobacter pseudethanolicus ATCC 33223 | TPD |
| Thermoanaerobacter tengcongensis MB4 | TTE |
| Thermobifida fusca YX | TFU |
| Thermosipho africanus TCF52B | TAF |
| Thermosipho melanesiensis BI429 | TME |
| Thermotoga lettingae TMO | TLE |
| Thermotoga maritima MSB8 | TMA |
| Thermotoga neapolitana DSM 4359 | TNE |
| Thermotoga petrophila RKU-1 | TPT |
| Thiomicrospira crunogena XCL-2 | TCX |
| Verminephrobacter eiseniae EF01-2 | VEI |
| Vibrio cholerae O1 eltor N16961 | VCH |
| Vibrio fischeri ES114 | VFI |
| Vibrio fischeri MJ11 | VFM |
| Vibrio harveyi ATCC BAA-1116 | VHA |
| Vibrio parahaemolyticus RIMD 2210633 | VPA |
| Vibrio splendidus LGP32 | VSP |
| Vibrio vulnificus CMCP6 | VVU |
| Vibrio vulnificus YJ016 | VVY |
| Xanthomonas axonopodis pv. citri 306 | XAC |
| Xanthomonas campestris pv. campestris ATCC 33913 | XCC |
| Xanthomonas campestris pv. vesicatoria 85-10 | XCV |
| Xanthomonas oryzae pv. oryzae KACC10331 | XOO |
| Xanthomonas oryzae pv. oryzae MAFF 311018 | XOM |
| Xanthomonas oryzae PXO99A | XOP |
| Xylanimonas cellulosilytica DSM 15894 | XCE |
| Yersinia enterocolitica subsp. enterocolitica 8081 | YEN |
| Yersinia pestis KIM | YPK |
| Yersinia pseudotuberculosis IP 32953 | YPS |
